# Observational study of a new strategy and management policy for measles prevention in medical personnel in a hospital setting

**DOI:** 10.1186/s12879-019-4139-4

**Published:** 2019-06-21

**Authors:** Chang-Pan Liu, Hsi-Peng Lu, Tainyi Luor

**Affiliations:** 1 Graduate Institute of Management, National Taiwan University of Science and Technology, Taipei, Taiwan; 2 Division of Infectious Diseases, Department of Internal Medicine, MacKay Memorial Hospital, Taipei, Taiwan

**Keywords:** Measles prevention, Medical personnel, Seropositivity, Vaccination coverage

## Abstract

**Background:**

At the end of March 2018, a clustered outbreak of measles associated with health care workers occurred in northern Taiwan. Prior to this study, the policy for measles vaccination for physicians and nurses in MacKay Memorial Hospital, Taiwan was encouragement of vaccination in medical personnel working in the emergency room or other high risk divisions without prior testing for measles antibody, and vaccination coverage was only 85.3%. It was important to urgently formulate a new strategy to achieve zero tolerance for intra-hospital transmission and epidemic prevention. This study aimed to explore the effectiveness of a new strategy for the prevention of an outbreak of measles.

**Methods:**

This study was conducted from April 23, 2018 to May 22, 2018 in the MacKay Memorial Hospital, a medical center and tertiary teaching hospital with 2200 beds in northern Taiwan. First-line medical personnel in the hospital underwent a free screening for measles antibody as a new strategy for measles outbreak prevention. Susceptible medical personnel were advised to receive measles vaccination.

**Results:**

A total of 719 first-line medical personnel were enrolled for the general survey. Measles seropositivity was 76.1% (287/377) in the generation born after 1978 (vaccinated), and 96.5% (330/342) in the generation born before 1978 (*p* < 0.001), while the overall seropositivity was 85.8% (617/719). Vaccination coverage of susceptible personnel under the new strategy reached 86.3% in the first month (88/102) following the survey. At the end of the first month after implementation of the new strategy, 98.1% of the medical personnel were seropositive or revaccinated, and reached 99.4% at the end of the second month.

**Conclusions:**

In this study, rapid, free antibody screening during a measles outbreak and subsequent vaccination of those susceptible resulted in most of the first-line medical personnel being seropositive or revaccinated. The new strategy was effective, time saving, used little manpower, and of low cost. Screening for measles antibody free of charge followed by vaccination of seronegative medical personnel can be regarded as an effective health management strategy to reduce and prevent the spread of measles infection.

## Background

Measles is a highly contagious disease [1–3]. Transmission of measles virus is primarily by airborne respiratory droplets [4], and can be severe and even fatal in infants, young children and the immunosuppressed [5, 6].

The basic reproduction number (*R*_0_) of measles is estimated to be 12–18, and can be even as high as 30.8 [7, 8]. The goal for measles vaccination coverage is typically > 95% of a population to achieve herd immunity [9, 10].

In Taiwan, routine measles vaccination for children was implemented since 1978; with a vaccination rate of > 97% [10]. The measles-mumps-rubella (M-M-R) vaccine is safe and well-tolerated [11], but some medical personnel at our hospital still refused vaccination for personal reasons.

In 1987 the United States was first country to establish a measles vaccine policy for health care personnel and the Advisory Committee on Immunization Practices (ACIP) report recommends that all health-care personnel have documentation of adequate vaccination against measles or other acceptable evidence of immunity [12–14]. Later, Australia, Canada, and some Caribbean and European countries implemented policies for measles vaccination in health care personnel. Measles vaccination was mandatory for medical personnel in Finland [13, 15], while in China vaccination was recommended, but not mandatory [15]. Measles vaccination is not mandatory in Taiwan for medical personnel, but highly recommended for those working in high risk divisions [10].

In order to assist countries to develop national policies for the vaccination of health care workers, the WHO recently updated and recommended worldwide standards and policies for measles vaccination. Accordingly, all health care workers and any staff who are in contact with patients should be immune to measles [16].

From 1989 to 2013, 53 worldwide published studies reported measles transmission from patients to medical personnel [13]. Among a staff of 890, 19 health care workers were infected with measles due to hospital-based transmission in Xinjiang Autonomous Region, China in 2016 [15]. Susceptible medical personnel are at higher risk of acquiring measles (estimated to be 2 to 19 times) and transmitting measles than the general population [13].

Although measles is not endemic in Taiwan, there are occasional import-linked cases, with 14 cases of measles reported in 2016, and 6 cases in 2017. However, from January 2018 to May 2018, 26 cases of measles were reported [10]. During this outbreak, three measles clusters were detected in Taiwan: linked to Tigerair Taiwan, in Chang Gung Memorial Hospital in northern Taiwan, and in a hospital in southern Taiwan.

In the measles cluster linked to Tigerair Taiwan, the index case was a male Taiwanese in his 30s who got measles infection in Thailand and travelled back to Taiwan. He then flew to Okinawa, Japan causing infection clusters in Taiwan and Japan [10].

The cluster of measles detected in Chang Gung Memorial Hospital, located near an airport in northern Taiwan in April 2018 included a male nurse in his 20s. Continuous monitoring was required for 491 people who had contact with him during the communicability period, including 13 nurses from the MacKay Memorial Hospital who studied with him in the same classroom.

On April 11, 2018, a woman in her 40s in northern Taiwan who had travelled to Hong Kong visited the emergency room and was admitted to a negative pressure isolation room in MacKay Memorial Hospital, and measles infection was subsequently confirmed [10]. The prevailing policy for infection control at our hospital was encouragement of vaccination for physicians and nurses working in high risk divisions (Divisions of Emergency Medicine, Infectious Diseases, Gynecology and Pediatrics) without prior antibody testing. Vaccination coverage under the former policy was 85.3% at our hospital.

Hence there was an urgent need to implement a new strategy to interrupt measles transmission for epidemic prevention. In the new strategy, first-line medical personnel were screened for measles antibody free of charge, and measles vaccination promoted for those who were seronegative to increase coverage. The goal was to achieve zero tolerance for intra-hospital transmission. This observational study aimed to explore the effectiveness of the new strategy to prevent the spread of measles in medical personnel.

## Methods

Priority for measles prevention was identification of seronegative first-line medical personnel and new employees. The new strategy involved screening for measles antibody free of charge in first-line medical personnel, following which those who were susceptible were advised to receive self-paid measles vaccination at cost price.

**Fig. 1:**
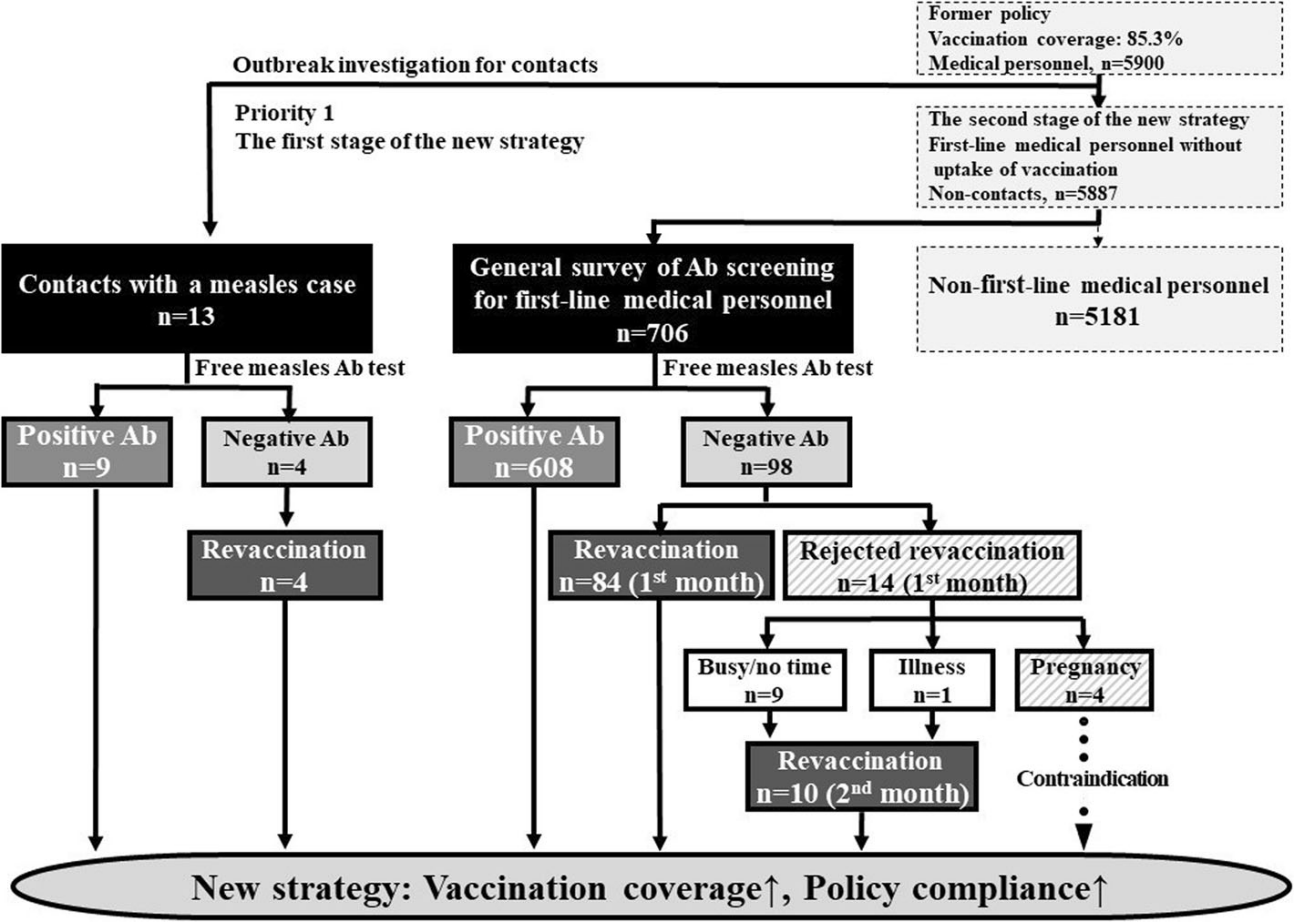
The flow chart of the research framework in the study (Ab, antibody)

We applied the health continuum care model “promotion, prevention, treatment, and recovery, PPTR model” [17, 18], integrated with the five stages of Rogers model of the innovation-decision process to the new strategy. The five stages of Rogers model of the innovation-decision process includes: (1) Knowledge (identification of contacts and antibody negative personnel) (2) Persuasion (promotion) (3) Decision (adoption or rejection) (4) Implementation (M-M-R vaccination of antibody negative personnel), and (5) Confirmation (collection of data on vaccination coverage and policy compliance) [17–19]. The research flow is shown in Fig. 1.

Compliance for the former policy describes the percentage of medical personnel that adhere to the former policy (vaccination for all first-line physicians and nurses at high risk of measles infection without prior antibody testing). Compliance for the new strategy describes the percentage of medical personnel that follow the new policy (implementation of measles immunoglobulin G testing for all first-line medical personnel and vaccination of seronegative personnel).

In the knowledge stage (identification of contacts and antibody negative personnel); prior conditions included unknown status of seropositivity in our medical personnel and occurrence of outbreak, while the innovative characteristic was testing for measles antibody which was rapid, convenient and free of charge.

In the persuasion stage (promotion), the priority was vaccination of those who were seronegative, with the advantage of preventing measles. Hence, an additional 1000 measles vaccines were urgently purchased at the start of the measles outbreak. In order to reduce the time taken to receive the antibody test results, the frequency for laboratory testing of measles antibody was increased from thrice a week to daily, from Monday to Saturday. After the biospecimen was obtained, it could be processed within 2 hrs. If the measles antibody test was performed in the morning, results were available by afternoon; so that those who were seronegative could visit the outpatient clinic and receive measles vaccination on the same day. The turnaround time for visiting the outpatient clinic after drawing blood was thus shortened from 3 days to 1 day, reducing complexity.

### Study population and clinical characteristics

This study was conducted in the MacKay Memorial Hospital, a medical center and tertiary teaching hospital with 2200 beds in northern Taiwan.

Among the 5900 employees in MacKay Memorial Hospital, the 13 nurses who had contact with the confirmed measles case at Chang Gung Memorial Hospital were immediately included in this study, and after free measles antibody testing, measles vaccination was arranged free of charge for the nurses who were seronegative.

Excluding 5181 non-first-line medical personnel, a total of 706 first-line medical personnel without a contact history and the 13 contacts were included in this study which was conducted from April 23, 2018 to May 22, 2018. These included medical and paramedical personnel in the following divisions: Emergency Medicine, Infectious Diseases, Gynecology, Pediatrics, Pulmonary Medicine, Otolaryngology, Family Medicine, Dermatology, Physiological Examination, Health Evaluation Center, Laboratory Medicine, Pharmacy, Radiology and Infection Control Center, and those working in the Negative Pressure Isolation Rooms. This study was approved by the institutional review board of MacKay Memorial Hospital (18MMHIS141).

Epidemiological data and clinical characteristics such as age, job description, division and exposure history were collected from the information system of MacKay Memorial Hospital. Measles antibody test was performed for these first-line medical personnel and the seropositivity rate was analyzed by age group.

### Measles antibody test

Measles antibody test was performed for detection of measles virus immunoglobulin G (IgG) (reference range of measles virus IgG: negative [< 13.5 arbitrary unit (AU)/mL], equivocal [13.5–16.4 AU/mL], positive [≥ 16.5 AU/mL]) using chemiluminescence immunoassay (CLIA) (LIAISON® Measles IgG assay with LIAISON®XL analyzer [DiaSorin, Saluggia, Italy]). The cutoff point of 16.5 AU/mL was set as the standard point for clinical decision. Values higher or equal to standard point were regarded as positive results, while lower values were regarded as negative.

### Statistical analysis

All analyses were performed using the Statistical Package for the Social Sciences (SPSS) software version 21.0 (SPSS Inc., Chicago, IL, USA). The chi-square test was used to analyze nonparametric data. For comparison within groups in which the expected number in any of the four cells was below five, a Fisher’s exact test was used. A *p* value < 0.05 was considered statistically significant.

## Results

The measles cluster in Chang Gung Memorial Hospital included a male nurse who had contact with 13 nurses working at our hospital. Continuous monitoring was required for 491 people who had contact with him during the communicability period, including these 13 nurses (Fig. 1).

Under the former policy, among the 462 physicians and nurses working in high risk divisions in our hospital with unknown antibody status, 68 refused vaccination, including 5 (one physician and four nurses) who worked in the high-risk Emergency Room.

Under the new strategy, the 13 nurses who had contact with the confirmed measles case underwent free measles antibody testing. Of these, 9 were seropositive. The knowledge stage was provision of free antibody screening for first-line medical personnel who had no contact history for measles during the outbreak. During the 1 month enrollment period, 706 first-line medical personnel were recruited. We conducted a screening test for measles antibody for these medical personnel free of charge (Fig. 1).

Among 102 seronegative medical personnel (4 in the first contact group, and 98 in the second universal group), 42.2% were nurses (43/102), 28.4% were paramedical personnel (29/102), 22.6% were administrative staff (23/102), and 6.9% were physicians (7/102).

**Fig. 2:**
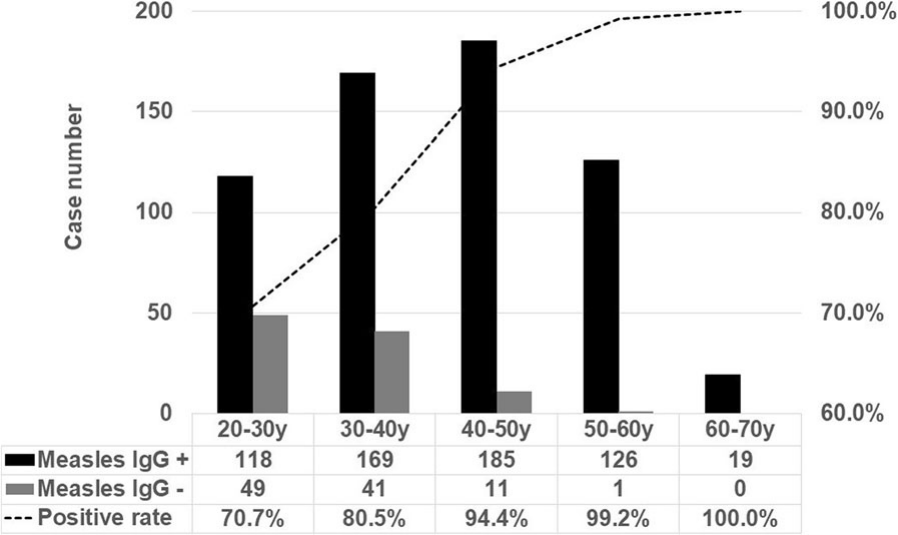
Histogram of positive/negative measles antibody rate in each age group

The implementation stage was M-M-R vaccination of antibody negative personnel. Four nurses who had contact with a case of measles were seronegative and susceptible for measles infection and were given priority for urgent measles vaccination free of charge. Excluding these four nurses, the other seronegative first-line medical personnel voluntarily received self-paid M-M-R vaccine at cost price. Overall measles seropositivity was 85.8% (617/719) at baseline. By age group, the seropositivity of measles antibody was 70.7% for those aged 20–30 years, 80.5% (169/210) for those aged 30–40 years, 94.4% (185/196) for those aged 40–50 years, 99.2% (126/127) for those aged 50–60 years, and 100% (19/19) for those aged 60–70 years (Fig. 2). The seropositivity of measles antibody was 76.1% (287/377) in the vaccinated generation, and 96.5% (330/342) in the unvaccinated generation (*p* < 0.001).

Finally, in the confirmation stage (collection of data on vaccination coverage and policy compliance), vaccination coverage reached 86.3% (88/102) in the first month under the new strategy. Reasons for refusal of vaccination were: too busy/no time (64.3%, 9/14), preparing for pregnancy or pregnant (28.6%, 4/14), and illness (7.1%, 1/14). At the end of the second month, only 4 medical personnel who had contraindications for vaccination had not yet been vaccinated, while the remaining seronegative medical personnel received vaccination. Vaccination coverage under the new strategy was 96.1% (98/102) at the end of the second month.

Compliance for the former policy and the new strategy were determined in the confirmation stage. At the end of first month after implementation of the new strategy, 98.1% (705/719) of the medical personnel were seropositive or revaccinated (88 were seronegative and received measles vaccination and 617 were seropositive), and reached 99.4% (715/719) in the second month.

In before-after comparison, compliance for the new strategy was higher than for the former policy (705/719 = 98.1% vs. 394/462 = 85.3%, *p* < 0.001). Furthermore, vaccination coverage under the new strategy was also higher than for the former policy (98/102 = 96.1% vs. 394/462 = 85.3%). It is notable that of the five emergency medical personnel who had previously refused vaccination under the former policy, two who were seronegative received measles vaccination immediately.

Cost-benefit analysis revealed that the total cost of universal vaccination for 719 first-line medical personnel was United States dollar (USD) $ 18,527 under the former policy of vaccination without testing for antibody. The overall cost for antibody testing and vaccination of seronegative personnel was USD $ 5675 under the new strategy, with a cost reduction of 69.4%. Finally, no medical personnel in our hospital were infected with measles during the outbreak.

## Discussion

The spread of measles has increased worldwide, with an increased number of cases been reported in Taiwan, Japan and Europe [10, 20]. We took advantage of the increased public awareness and heightened alarm in the general public caused by the current measles outbreak to devise a new strategy for epidemic prevention which was rapid and effective.

Prior to the implementation of the new strategy, only 85.3% (394/462) of our medical personnel were seropositive or had measles vaccination. There are very few laws regulating vaccine administration for medical personnel, including penalties for non-compliance. The presence of a national measles vaccine policy for medical personnel does not guarantee implementation [13]. Thus, it is very important to adopt measures to increase vaccine coverage such as offering free measles antibody testing, increasing the convenience and speed of testing for measles antibody and promoting vaccination of susceptible personnel, especially during an outbreak of measles.

In the confirmation stage, the new strategy was not only effective in outbreak prevention but was also economical, thus reinforcing the innovation-decision process for its adoption.

On April 242,018, the Taiwan CDC (Centers for Disease Control) issued an emergency announcement concerning measles vaccination policy due to a shortage of measles vaccine in Taiwan, with priority given to those at high risk of measles infection. Measles vaccination would be made available to the general population if there were adequate stocks remaining.

During this period, our hospital had a surplus of measles vaccine due to early purchase at the start of the outbreak, and self-paid vaccines were available to the public who visited the outpatient clinics of MacKay Memorial Hospital seeking urgent vaccination. Thus beneficial health care service was provided during the period of vaccine shortage. In the event of a measles outbreak, it is not essential to provide compulsory free vaccination to all the medical personnel; only the seronegative, susceptible personnel require vaccination, reducing complexity and difficulty.

In the last decade, Dominguez et al. found that an outbreak of measles occurred in non-vaccinated persons, mainly those ≤15 months even in an area with a high vaccination coverage rate [9]. Concerning measles vaccination in Taiwan, two doses of M-M-R vaccine are recommended. It was estimated that vaccine coverage was 97% in Taiwan [21, 22]. However, measles seropositivity rate for medical personnel in the vaccinated era was only 76.1% (287/377) compared to 96.5% (330/342) in the pre vaccination era.

In a general survey conducted by Chen at al. on 3552 subjects in 2007 from northern, central and southern Taiwan, the overall seropositive rate of measles was 74.7% [21]. The antibody positivity rate after measles vaccination in Taiwan showed a decreasing trend with age in the vaccinated generation. Although decreasing seropositivity after measles vaccine does occur, two-dose vaccination is generally thought to be adequate to eliminate measles infection [23]. Testing for antibody is often used to evaluate immunity after measles vaccination. However, T cell immune response is also important for long term protection against measles infection [24].

Measles antibody positivity was 74.7% in Taiwan [21], 80% in Japan [25] and 85.8% at baseline in this study. In a study conducted between 2004 and 2009 on health care workers at a hospital in southern Taiwan, Ho et al. found that measles antibody positivity was 78.1% for the age group 20–29 years [26]. However, measles seropositivity for the age group 20–29 years in first-line medical personnel in our hospital in 2018 was even lower than reported by Ho et al.

In the past, some medical personnel at our hospital were unconcerned and did not know whether they had serologic protection for measles, hence they continued to reject vaccination. We took advantage of the occurrence of the measles outbreaks to adopt a new strategy for epidemic prevention. The new strategy was to provide measles antibody testing which was rapid, convenient and free of charge. Since measles is a highly contagious disease [1–3, 27], the presence of an outbreak increased the fear for measles infection and resulted in increased willingness of our medical personnel to receive vaccination. The rate of medical personnel who were seropositive or revaccinated reached 98.1% at the end of the first month and was 99.4% at the end of the second month following implementation of the new strategy.

The new strategy was effective in encouraging first-line susceptible seronegative medical personnel to receive measles vaccination, thus increasing vaccination coverage. Implementation of this new strategy in other hospitals could further reduce the spread of measles and may even be applied to other infectious diseases for the prevention of outbreaks.

However, the potential risk for measles infection remains because of the possibility of primary and secondary measles vaccine failure in a small percentage. Failure to develop protective antibody levels in those immunized with two doses of measles vaccine (primary failure), and waning immunity over time (secondary failure) may result in measles infection [13, 28].

Taiwan has maintained a vaccination coverage of greater than 95–97% with two-dose of M-M-R vaccine in the preschool stage [21, 22], but 3 clusters of measles still occurred in 2018. Hence in addition to vaccination, continuous vigilance and surveillance for early detection of cases and other infection control measures are also crucial in preventing the spread of measles. Due to the success of the new strategy, extension of free measles antibody testing to include all personnel (medical and administrative) in our hospital, with encouragement of seronegative personnel to receive measles vaccination is being considered. This would ensure that all the hospital staff has immunity against measles, as diagnosis of primary measles cases is often delayed.

### Study limitation

This study was conducted at a single medical center over a short period. Further long term studies conducted in different hospitals could provide more data regarding the effectiveness of the new strategy in interrupting the spread of measles and preventing an outbreak in a larger population.

## Conclusions

Rapid and free antibody screening for first-line medical personnel during a measles outbreak followed by vaccination of susceptible individuals increased vaccination coverage, helping to prevent the spread of measles. The new strategy was effective (no medical personnel were infected with measles during the outbreak), time saving (1 to 2 months), used little manpower (screening only for high risk first-line medical personnel), and of low cost. Screening for measles antibody free of charge followed by vaccination of seronegative medical personnel can be regarded as an effective health management strategy to reduce and prevent the spread of measles infection.

## Data Availability

The database used in this study is available upon request sent to the corresponding author.

## Abbreviations

Ab: Antibody
AU: Arbitrary unit
CDC: Centers for Disease Control
CLIA: Chemiluminescence immunoassay
IgG: Immunoglobulin G
M-M-R vaccine: Measles-mumps-rubella vaccine
PPTR: Promotion, prevention, treatment, recovery
*R*_0_: Basic reproduction number
SPSS: Statistical Package for the Social Sciences
USD: United States dollar
WHO: World Health Organization
